# *Ac*-HSP20 Is Associated With the Infectivity and Encystation of *Acanthamoeba castellanii*

**DOI:** 10.3389/fmicb.2020.595080

**Published:** 2021-01-12

**Authors:** Ningning Wang, Hongyu Sun, Di Liu, Xiaoming Jiang, Meiyu Zheng, Wenhe Zhu, Quan Liu, Wenyu Zheng, Xianmin Feng

**Affiliations:** ^1^Department of Pathogenic Biology, Jilin Medical University, Jilin City, China; ^2^Department of Microsurgery, Jilin City Central Hospital, Jilin City, China; ^3^School of Life Sciences and Engineering, Foshan University, Foshan, China

**Keywords:** Acanthamoeba, heat shock protein-20, encystation, infectivity, Acanthamoeba keratitis

## Abstract

*Acanthamoeba castellanii* is a pathogenic and opportunistic free-living amoeba that causes *Acanthamoeba* keratitis (AK) and granulomatous amebic encephalitis (GAE) in immunocompromised individuals. The biological and pathogenic characterizations behind this opportunistic protozoan is not fully understood. This study aimed to determine the biological functions of heat shock protein (HSP)-20 of *A. castellanii* (*Ac*-HSP20) involved in the maintenance of life cycle and the infectivity of *A. castellanii*. Immunoscreening *A. castellanii* cDNA library with *A. castellanii* infected rabbit sera identified three positive clones, one of them was a putative heat shock protein (*Ac*-HSP20). The recombinant 23 kDa *Ac*-HSP20 protein (r*Ac*-HSP20) was successfully expressed in *Escherichia coli* BL21 (DE3) and purified using metal affinity chromatography. The rabbits immunized with r*Ac*-HSP20 produced high titer antibody (1:25,600). Immunolocalization with the antibody identified the expression of native *Ac*-HSP20 on the surface of both *A. castellanii* trophozoites and cysts. Further, Western blot with antibody identified that the expression of native Ac-HSP20 was 7.5 times higher in cysts than in trophozoites. Blocking *Ac*-HSP20 on the membrane of trophozoites with specific antibody or silencing *Ac-hsp20* gene transcription by siRNA inhibited their transformation into cysts at the early stage but returned to normal at the late stage by stimulating the transcription of *Ac-hsp20*. Incubation of trophozoites with anti-*Ac*-HSP20 IgG increased macrophage-involved phagocytosis to the protozoa and inhibited trophozoite infectivity on the cornea of rabbits compared with that without antibody. Our study provides that *Ac*-HSP20 is a surface antigen involved in the encystation and infectivity of *A. castellanii* and thus an important target for vaccine and drug development.

## Introduction

*Acanthamoeba castellanii* is a free-living protozoan that can cause *Acanthamoeba* keratitis (AK) and granulomatous amebic encephalitis (GAE) in immunocompromised individuals. AK is a rare and severe disease in which AK trophozoite adheres and infects the cornea epithelial cell layer and the paracentral corneal stroma, thereby causing permanent visual impairment (Clarke and Niederkorn, 2006; Khan, 2006; Siddiqui and Khan, 2012). The clinical signs of AK include photophobia, annular matrix infiltration, epithelial defect, and orbital edema. GAE is a life-threatening infection in central nervous system (CNS). It usually occurs in immunocompromised individuals with solid organ transplantations or patients with AIDS (Marciano-Cabral and Cabral, 2003). People become infected through direct contact with contaminated water. The amoebae invade skin, sinuses, or lungs and then reach the CNS through hematogenous route to cause GAE and even death (Parija et al., 2015).

*Acanthamoeba castellanii* have two stages in its life cycle, including vegetative trophozoite and dormant cyst. Trophozoites usually live in soil and water in the environment fed on bacteria. When external environmental conditions are not suitable for amoeba growth, such as extreme changes in temperatures, pH, osmolarity, and desiccation, the trophozoite transforms into cyst, which is a self-protective form. When stress conditions are removed, the cyst can reverse back to its trophozoite form. Studies have shown that trophozoites are more susceptible to antimicrobial agents than cysts (Lorenzo-Morales et al., 2013; Mahboob et al., 2020), but cysts are resistant to chemicals and could survive in the environment and maintain their pathogenicity for several years. Trophozoites are related to the adhesion and invasion causing diseases, while cysts are related to chronicity, drug resistance, recurrence, and transmission of the infection (Turner et al., 2000, 2004). Meanwhile, cysts could survive several years as a source of recurrent transmission. Therefore, preventing trophozoites from being converted to cysts is important to the treatment of *A.castellanii* infections and blocking its transmission.

Given the difficulty in diagnosing *Acanthamoeba*-caused AK and GAE and the serious consequences of the infection (Visvesvara, 2010), it is needed to develop a vaccine especially for those with immunocompromising conditions. In an effort to identify antigens that are immunogenic and protective as vaccine candidates, a serum from rabbit infected with *A. castellanii* in its corneal stroma was used in the present study to immunoscreen a cDNA library of *A. castellanii* trophozoites. One of the positive clones encoded the heat shock protein (HSP)-20 of *A. castellanii* (*Ac*-HSP20). HSPs are a group of highly conserved proteins found in all organisms that primarily function as molecular chaperones (Lindquist and Craig, 1988). In accordance with their molecular weight, the proteins in HSP superfamily are classified into eight major sub-families: HSP110, HSP100, HSP90, HSP70, HSP60, HSP40, HSP10, and small HSP (sHSP; Feder and Hofmann, 1999). HSP20 is one of the sHSPs with the characteristics of the sHSP family. When cells are under stress, sHSPs could bind to denatured proteins and maintain a competitive folding state to prevent their irreversible aggregation (Sun and MacRae, 2005; Nakamoto and Vígh, 2007). sHSPs play important roles in maintaining life cycle and the pathogenicity of parasites. Knockout of HSP20 in *Plasmodium berghei* affected the substrate-dependent cell movement of sporozoites and reduced sporozoite-matrix adhesion and the spread of natural malaria (Montagna et al., 2012). In *Leishmania* protozoa, the deletion of HSP23 reduces their viability in harsh environment with chemical stressors, such as ethanol and semimetal ions, and loses the ability to infect macrophages (Hombach et al., 2014).

In the present study, we reported the cloning of *Ac*-HSP20 at the first time and its functions in encystation and infectivity of *A. castellanii*. The results showed that this protein is one of the immunodominant antigens and plays a vital role in the maintenance of *Acanthamoeba*’s life cycle.

## Materials and Methods

### *A. castellanii* Cultivation

*Acanthamoeba castellanii* cysts were obtained from ATCC (ATCC 30011) and cultured in peptone-yeast extract-glucose medium containing 50 μg/ml of gentamicin, pH 6.5, in a cell culture flask at 25°C to transform into trophozoite form. The cultured trophozoites were fed with heat-killed *Escherichia coli* (ATCC 29552) and harvested at their logarithmic growth phase 3–5 days after cultivation. The cysts were harvested after cultivation for 10–14 days.

### Animals

New Zealand white rabbits (8–10 months old weighing 2.5–4 kg) were purchased from the Animal Facility of Jilin University (China). The experimental protocols were approved by the Animal Care and Use Committee of the Jilin Medical College (approval number: 190001). The animal care and treatment in this study followed the statement of the Association for Research in Vision and Ophthalmology.

### Rabbit Peritoneal Macrophages

One rabbit was injected with 200 ml of LB broth into the peritoneal cavity, and the cavity liquid was retrieved 3 days after the injection. The macrophages were isolated from the retrieved cavity liquid by adhering on culture flasks. The obtained rabbit macrophages were maintained in RPMI 1640 medium supplemented with 10% fetal bovine serum and 50 μg/ml of streptomycin, 100 U/ml of penicillin, and 50 μg/ml of gentamicin in a cell culture flask at 5% CO_2_, 37°C.

### Cloning of *Ac*-HSP20

One rabbit was infected with 1 × 10^4^ *A. castellanii* trophozoites through micro-injection into the eye stroma to induce AK. The serum was obtained to immunoscreen the cDNA library *A.castellanii* trophozoite as described before (Wang et al., 2014; Feng et al., 2015). The DNA was extracted from the positive clones for double-stranded DNA sequencing. The obtained DNA sequences were aligned with sequences deposited in GenBank through Basic Local Alignment Search Tool (BLAST) search to conclude their homologs. One of the three positive clones shared 59% amino acid sequence identity with a Hsp20/alpha crystallin superfamily protein of *A. castellanii* (GenBank accession: XP_004336746.1; Zhang et al., 2018), thus named as *Ac*-HSP20 at the first time.

### Expression and Purification of Recombinant *Ac*-HSP20 and Preparation of Polyclonal Antibodies

The DNA encoding for *Ac*-HSP20, synthesized in Beijing Genomics Institute, was subcloned into pET-22b expression vector (New England Biolabs, Beijing, China) by using BamHI and HindIII sites. The sequencing-confirmed recombinant pET-22b-*Ac*-HSP20 plasmid was transformed into *E. coli* BL21 (DE3) competent cell (Beyotime, Beijing, China). The recombinant *Ac*-HSP20 protein (r*Ac*-HSP20) was expressed under the induction of 1 mM of IPTG. The expressed r*Ac*-HSP20 with His-tag at C-terminus was purified *via* nickel affinity and DEAE ion-exchange chromatography. The anti-*Ac*-HSP20 serum was produced by immunizing a rabbit with 400 μg of r*Ac*-HSP20 emulsified with complete Freund’s adjuvant and boosted twice with 200 μg of r*Ac*-HSP20 emulsified with incomplete Freund’s with 2-week interval. The anti-*Ac*-HSP20 IgG was purified from the antiserum through a HiTrap Protein A HP column (GE Healthcare, United States). The antibody titer of the purified polyclonal antibody was measured using ELISA.

### Immunofluorescence Assay

The fresh trophozoites of *A. castellanii* were washed with the pH 7.4 phosphate buffered saline (PBS), overlaid on a coverslip pretreated with 1 mg/ml of poly-L-lysine, and then fixed with 3% paraformaldehyde, followed by permeabilization with 0.25% Triton X-100. The protozoa were incubated with rabbit anti-*Ac*-HSP20 IgG (20 μg/ml) or normal rabbit serum (1:50) and then with Alexa Fluor 488-conjugated anti-rabbit IgG secondary antibody (1 μg/ml, Abcam, United States). The trophozoites were counterstained for the nucleus with 1 μg/ml of 4',6-diamino-2-phenylindole and stained for the cytomembrane with 10 umol 1,1'-dioctadecyl-3,3,3',3'-tetramethylindocarbocyanine perchlorate (DiI). Images were captured with a laser scanning confocal microscope (Olympus, Tokyo, Japan).

### Macrophage Phagocytosis of *A. castellanii* Trophozoites

Freshly collected 1 × 10^5^ *A. castellanii* trophozoites were incubated with the same number of macrophages collected from the rabbit peritoneal cavity in the presence of different amounts of rabbit anti-*Ac*-HSP20 IgG (40, 20, 10, 5, 2.5, and 1.25 μg/ml). Normal rabbit serum was used as the control (1:50 dilution). The cells were incubated in a CO_2_ incubator at 37°C for 2 h. The change in macrophage phagocytosis of the trophozoites was observed under the microscope. The macrophage phagocytosis rate was calculated by courting the number of macrophages with engulfed trophozoites.

### Rabbit Corneal Infection With *A. castellanii*

Eight New Zealand rabbits were randomly divided into two groups with four rabbits each. The eyes of all rabbits were immunosuppressed with 0.5% hydrocortisone ophthalmic solution drops four times daily for 3 consecutive days before exposure to *A. castellanii* trophozoites. In the experimental group, the right eyes of four rabbits were inoculated with 200 μl of 2 × 10^5^ *A. castellanii* trophozoites preincubated with rabbit anti-*Ac*-HSP20 IgG (40 μg/ml) at 37°C for 30 min. In the control group, the right eyes of four rabbits were infected with the same number of trophozoites without incubation with antibody. The left eyes of all rabbits were dropped with 200 μl of sterile saline as the normal control. Corneal scraping was performed on days 3, 7, 14, and 28 after the infection, the living trophozoite number was counted under three randomly-picked fields, and the average was calculated to determine the intensity of corneal infection.

### The Expression of *Ac*-HSP20 in *A. castellanii* Trophozoites and Cysts

Freshly collected *A. castellanii* trophozoites were incubated with rabbit anti-*Ac*-HSP20 IgG (40 μg/ml) for 30 min before being placed on ice for 0, 6, 12, and 24 h. In control group, the same numbers of trophozoites were directly put on ice without incubating with antibody. The transformation rate from trophozoite to cyst at different time points was measured and counted under the microscope.

The transcriptional expression levels of *Ac-hsp20* at different cooling time points were measured with real-time quantitative PCR (qPCR). The total RNAs of *A. castellanii* trophozoites or cysts at different time points were extracted using an RNA extraction kit (Beyotime, Beijing, China). The concentration of RNA was measured at A260 and the purity was checked at A260/280. The quality and integrity of RNA were assessed on 1% formaldehyde-agarose gel. The total RNA was treated with DNase I and then reverse transcribed into cDNA by using Oligo dT primers. The transcriptional level of *Ac-hsp20* was quantitatively measured using qPCR (NovoStart SYBR qPCR SuperMix Plus, Novoprotein, Shanghai, China) with specific primers 5'-AAGGCGAGAACTGGGTGA-3' (forward) and 5'-CGGGCTTGGGTACTACAAT-3' (reverse). The beta-actin (β-actin) housekeeping gene was measured as the control by using primers 5'-GTATGCTCCTCCTCAAG-3' (forward) and 5'-TAGAAGGTGTCCATCCA-3' (reverse). All qPCR assays were conducted in an ABI 7500 fast real-time PCR system (ABI7500 Fast, United States).

To determine the protein expression levels of native *Ac*-HSP20 at the different stages of *A. castellanii*, total proteins were extracted from 1 × 10^8^ trophozoites or cysts by suspending in cell lysis buffer containing 10 mM of PMSF for 30 min and sonicating for several times on ice. The lysates were centrifuged at 12,000 rpm for 10 min, and the supernatants were collected as *A. castellanii* trophozoite or cyst extracts for Western blot with anti-*Ac*-HSP20 IgG.

For Western blot, the same amount of trophozoite or cyst extracts (40 μg) was run on SDS-PAGE and then transferred on polyvinylidene fluoride (PVDF) membrane. The membrane was recognized using rabbit anti-*Ac*-HSP20 IgG (2 μg/ml). Meanwhile, the same amount of extracts was recognized using rabbit anti-β-actin serum (1 μg/ml, Bioss, Beijing, China) as the comparative control. Normal rabbit serum was used as negative control. HRP-conjugated goat anti-rabbit IgG were used as secondary antibody (Bioss, Beijing, China). The blots were visualized using ECL chemiluminescence reagents (Beyotime, Beijing, China) and imaged on a luminescence imaging system (Tanon, Beijing, China).

Silence of *Ac-hsp20* expression with siRNA to silence the *Ac-hsp20* gene expression, the siRNA targeting *Ac-hsp20* was designed and performed by GenePharma (Shanghai, China). The siRNA duplex with sense (5'GACUGGUCAGCUAGCGAGAUTT-3') and anti-sense (5′UGAUCUCGCUAGCUGACCAGCTT-3') sequences were synthesized and conjugated with FITC. The siRNA was added into 1 × 10^6^ *A. castellanii* trophozoites to 200 nM in total volume of 2.5 ml PYG media. The siRNA duplex with sense 5'-UUCUCCGAAC GUGUCACGUTT-3') and anti-sense (5'-ACGUACACGUUCGGAGAATT-3') was used for negative control (NC). After being incubated for 6 h, the trophozoites were washed with PYG medium and the intake of siRNA in the trophozoites was observed and imaged under a fluorescence microscope. Twelve hours after the incubation, qPCR was performed to measure the *Ac-hsp20* mRNA transcription level, and *Ac*-HSP20 protein expression level was performed by Western blot as described above 48 h after the siRNA transfection. To investigate the role of *Ac*-HSP20 in the encystation of *A. castellanii* trophozoite, siRNA transfection was performed in trophozoites. Twelve hours after siRNA transfection, the trophozoites were incubated on ice and the encystation rate was counted under microscope 0, 6, 12, and 24 h after being incubated on ice.

### Statistical Analysis

GraphPad Prism8 software was used to analyze the experimental data. The results were expressed as mean ± standard deviation; statistical analysis was performed using one-way or two-way ANOVA to determine the significance of the difference between two groups. *p* < 0.05 indicated statistically significant difference (^*^p < 0.05, ^**^p < 0.01, and ^***^p < 0.001).

## Results

### Cloning of *Ac*-HSP20

Three positive clones were obtained after screening 2 × 10^5^ colonies of *A. castellanii* trophozoite cDNA expression library with serum of rabbit infected with *A. castellanii* in its corneal. One of the positive clones shared 59% amino acid sequence identity with HSP20/alpha crystallin superfamily protein of *A. castellanii* (GenBank accession: XP_004336746.1), thus a new putative HSP20 family protein, named as *Ac*-HSP20. The sequence of *Ac*-HSP20 was submitted to GenBank, with accession number MT323119.

### Expression of Recombinant *Ac*-HSP20

**Figure 1:**
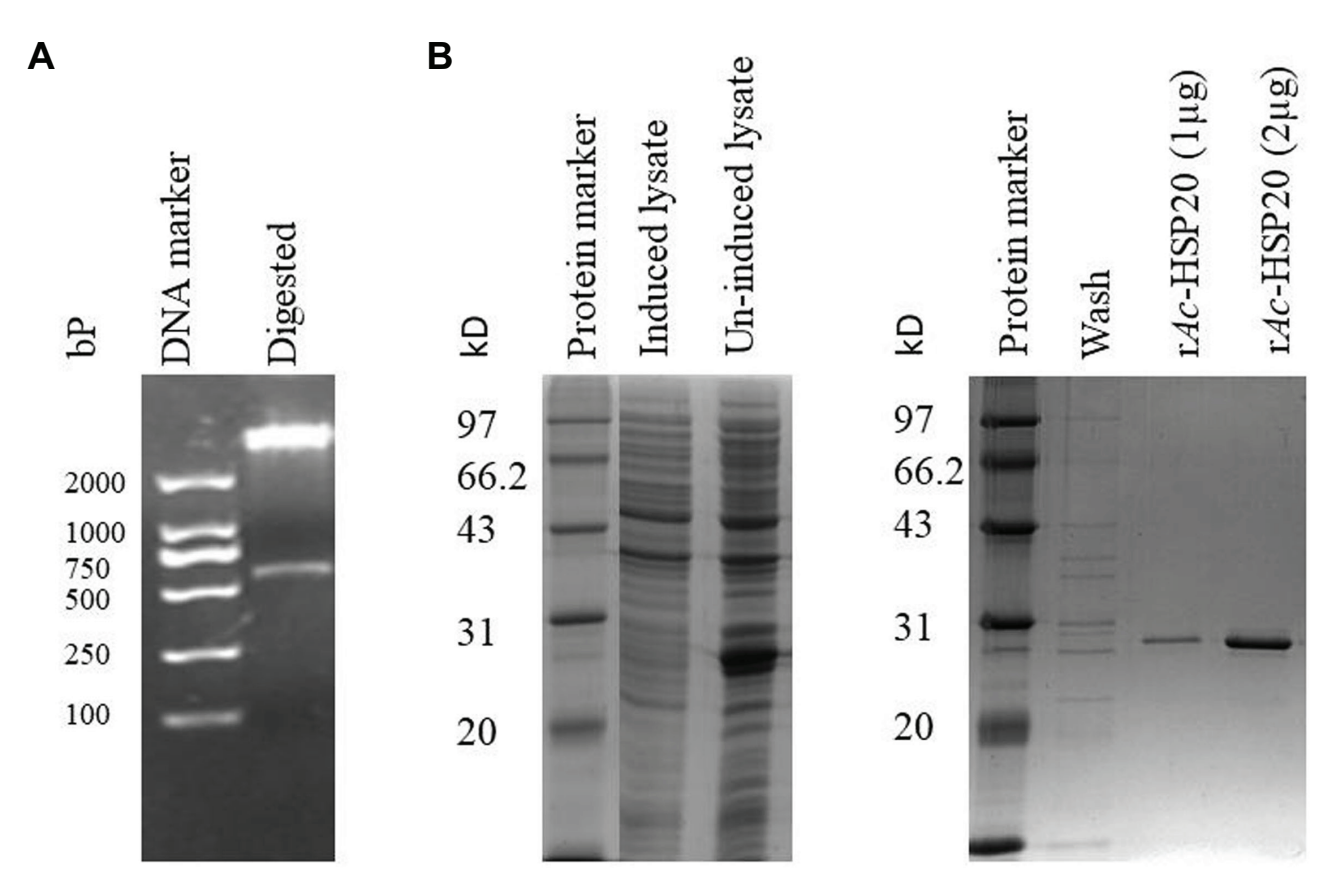
Cloning and expression of recombinant heat shock protein (HSP)-20 of *Acanthamoeba castellanii* (*Ac*-HSP20) in *Escherichia coli* BL21 (DE3). Agarose gel electrophoresis showing a 583 bp insert digested with *Bam*HI and *Hind*III from *Ac-hsp20*/pET22b plasmid DNA **(A)**; SDS-PAGE of r*Ac*-HSP20 expressed in *E. coli* BL21 and the purified r*Ac*-HSP20 (2 μg; **B**).

The 583 bp DNA coding for full-length *Ac*-HSP20 was successfully subcloned into pET22b (Figure 1A). The recombinant plasmid DNA was transformed into *E. coli* BL (DE3), and *Ac*-HSP20 was expressed as 26 kDa soluble recombinant protein (r*Ac*-HSP20) in the bacteria under the induction of 1 mM IPTG. The r*Ac*-HSP20 with His-tag was purified using nickel affinity chromatography (Figure 1B).

### *Ac*-HSP20 Localization on the Membrane of *A. castellanii* Trophozoite and Cyst

**Figure 2:**
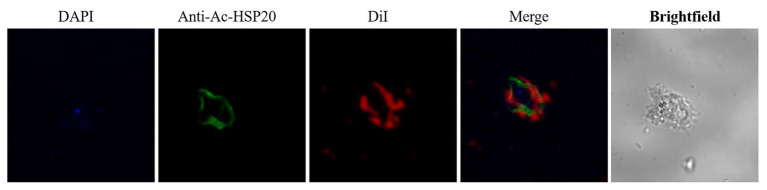
Immunolocalization of *Ac*-HSP20 in *A. castellanii* trophozoite and cyst detected by IFA with anti-*Ac*-HSP20 IgG (20 μg/ml) and visualized by Alexa-Fluor488 labled anti-rabbit IgG secondary antibody (1 μg/ml). Normal rabbit serum (1:50) was used as control. Nuclei were stained with DAPI, membrane was stained with DiI. Scale bars: 10 μm.

To determine the subcellular localization of *Ac*-HSP20 in *A. castellanii* protozoa, the rabbit anti-*Ac*-HSP20 serum was raised in rabbit and the purified anti-*Ac*-HSP20 IgG was used to recognize native *Ac*-HSP20 expressed on *A. castellanii* trophozoite using IFA. IFA results showed that *Ac*-HSP20 was mainly localized on the cell membrane of *A. castellanii* trophozoite (Figure 2).

### Anti-*Ac*-HSP20 Antibody Increased Macrophage Phagocytosis on *A. castellanii* Trophozoites

**Figure 3:**
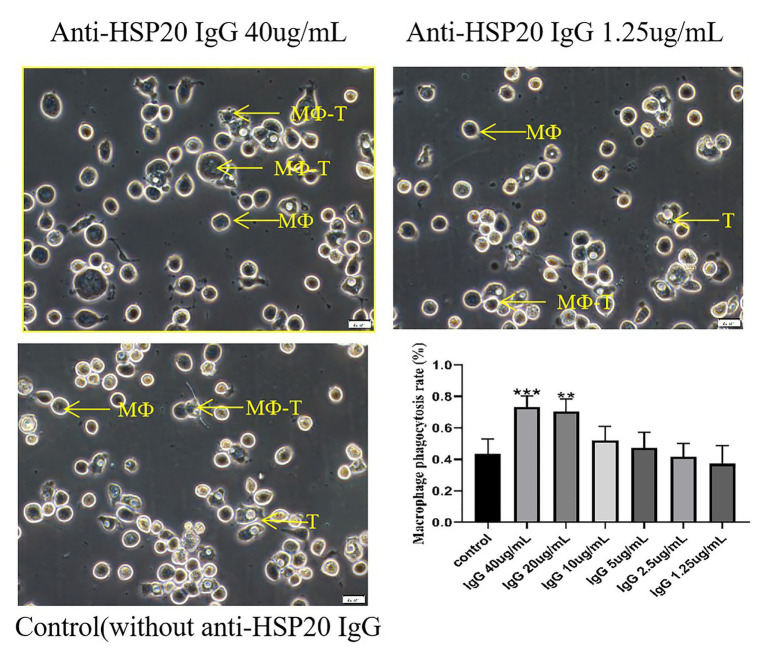
*Ac*-HSP20 antibody induced macrophage phagocytosis on *A. castellanii* trophozoites at a IgG amount-dependent pattern. The phagocytosis of macrophages on *A. castellanii* trophozoites after co-incubation with anti-Ac-HSP20 IgG at 40 μg/ml and 1.25 μg/ml, or normal rabbit serum (1:50) as control, was observed under microscope. The macrophage phagocytosis rate at different amount of anti-*Ac*-HSP20 IgG was shown at downright penal. ^***^p < 0.001, ^**^p < 0.01, compared with normal control. MФ, macrophage; T, trophozoites; MФ-T, macrophages with trophozoite phagocytized. Scale bars:20 μm.

The macrophages were collected from rabbit peritoneal cavity and incubated with the same number of *A. castellanii* trophozoites in the presence of different amounts of rabbit anti-*Ac*-HSP20 IgG (40, 20, 10, 5, 2.5, and 1.25 μg/ml) to determine whether anti-*Ac*-HSP20 antibody induced antibody-dependent macrophage phagocytosis on *A. castellanii*. The rate of macrophage phagocytosis (containing *A. castellanii* trophozoite intracellularly) was 73.1% when the antibody concentration was 40 μg/ml. The phagocytosis dropped to 37.2% when the antibody concentration was decreased to 1.25 μg/ml, which was similar to the culture with addition of or normal rabbit serum (1:50), with statistical significance among the difference antibody amount (^***^p < 0.001 and ^**^p < 0.01, Figure 3). These results demonstrated that anti-*Ac*-HSP20 IgG induced macrophage phagocytosis on *A. castellanii* trophozites.

### Role of *Ac*-HSP20 in the Infectivity of *A. castellanii* Trophozoite to Rabbit Cornea

**Figure 4:**
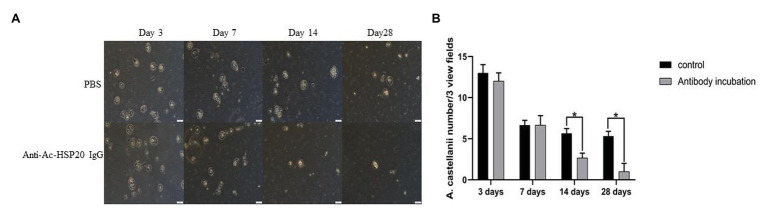
Observation of *A. castellanii* trophozoite on corneal scraping of rabbits after being infected with 2 × 10^5^ *A. castellanii* trophozoites incubated with PBS or with rabbit anti-*Ac*-HSP20 IgG (40 μg/ml) on day 3, 7, 14, and 28 after infection under microscope **(A)**. The average counted number of trophozoites per three view fields at different day points (**B**; ^*^p < 0.05). Scale bar: 20 μm.

The right eyes of four rabbits were infected with 2 × 10^5^ *A. castellanii* trophozoites, while another group of four rabbits was infected with the same number of trophozoites preincubated with rabbit anti-*Ac*-HSP20 IgG (40 μg/ml) on their right eyes. Corneal scraping was performed on days 3, 7, 14, and 28 after the infection, and the living trophozoites were counted on the corneal scrapes. The protozoan counting results showed no difference in the living protozoan number between the two groups on days 3 and 7. However, the living protozoan number was significantly reduced on the cornea of rabbits infected with *A. castellanii* trophozoites incubated with anti-*Ac*-HSP20 antibody at late stages (days 14 and 28) compared to the group of rabbits infected with protozoan without antibody (*p* < 0.05, Figure 4). No protozoan was observed on the left eyes of rabbits that received sterile saline in each group.

### Predominant Expression of *Ac*-HSP20 Protein in *A. castellanii* Cysts

**Figure 5:**
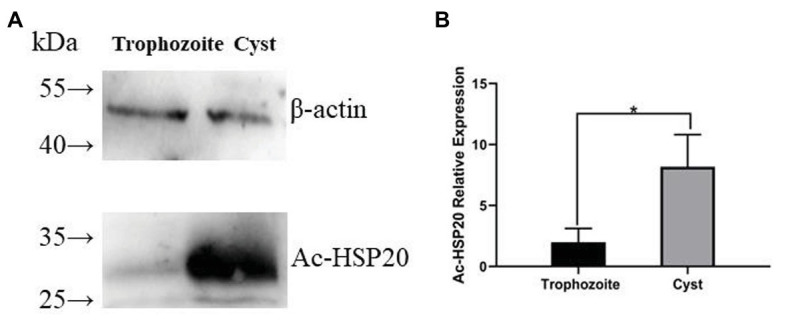
The predominant expression of *Ac*-HSP20 in cysts of *A. castellanii* determined by Western blot with anti-*Ac*-HSP20 IgG. Total 40 μg of *A. castellanii* trophozoite and cyst extracts was transferred on polyvinylidene fluoride (PVDF) membrane, recognized by rabbit anti-*Ac*-HSP20 IgG (2 μg/ml). The same amount of protozoan extracts was equally recognized by anti-β-actin as control **(A)**. Statistical analysis of the predominant expression of *Ac*-HSP20 in cysts of *A. castellanii* (^*^p < 0.05; **B**).

The same amount of trophozoite and cyst extracts was transferred on PVDF membrane and recognized using the rabbit anti-*Ac*-HSP20 IgG to further determine the expression of native HSP20 protein in different forms of *A. castellanii*. Western blot results showed that the expression of native *Ac*-HSP20 was 7.5 times higher in cysts than in trophozoites (*p* < 0.05), while the control β-actin was the same in both forms of *A. castellanii* (Figures 5A,B). Both trophozoite and cyst extracts were not recognized by normal rabbit serum. These results indicated that *Ac*-HSP20 was predominantly expressed in cyst and possibly essential for the encystation of *A. castellanii*.

### Role of *Ac*-HSP20 in the Encystation of *A. castellanii* Trophozoite

**Figure 6:**
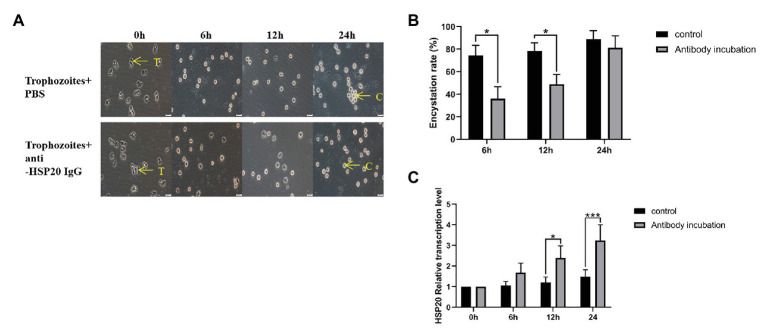
Effect of *Ac*-HSP20 on the encystation of *A. castellanii* trophozoites. The trophozoites were incubated with 40 μg/ml rabbit-anti-*Ac*-HSP20 IgG or PBS control and then cooled down on ice for 0, 6, 12, and 24 h. The transformation from trophozoites to cysts was observed under microscope **(A)**. The encystation rate of *A. castellanii* trophozoites at different ice incubation time points was calculated **(B)**. The *Ac-hsp20* gene transcription level in protozoa at different cooling time points was measured by quantitative PCR (qPCR) compared to trophozoites without antibody incubation (^*^p < 0.05, ^***^p < 0.001; **C**). T, trophozoites; C, cysts. Scale bars: 20 μm.

Trophozoites typically begin to transform to cysts after being cooled down on ice, most of them transformed into cysts within 24 h. However, after being incubated with 40 μg/ml of rabbit anti-*Ac*-HSP20 IgG, the cyst transformation was significantly reduced at the early time points (6 and 12 h) compared with group without antibody (PBS). However, no significant difference in cyst transformation was noted between the antibody and PBS groups at 24 h time point, with most trophozoites transformed into cysts at both groups (Figures 6A,B). However, gene transcription results showed that incubation with anti-*Ac*-HSP20 IgG significantly increased the *Ac-hsp20* gene transcription level upon temperature cooling down compared with protozoa without antibody incubation (^*^p < 0.05 and ^**^p < 0.001, Figure 6C).

**Figure 7:**
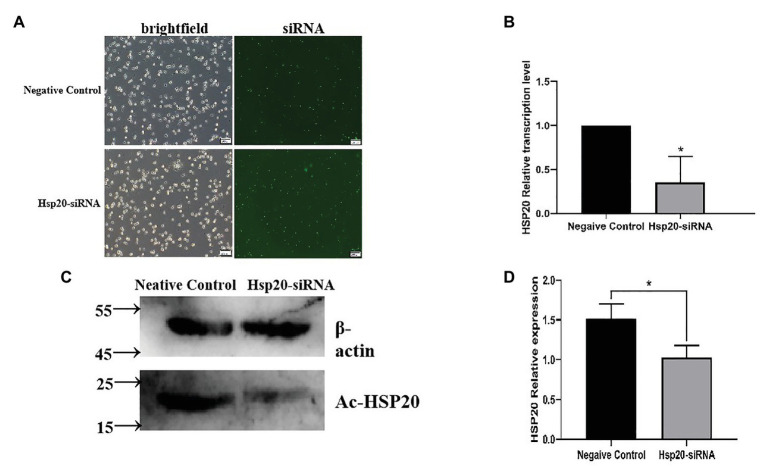
The *Ac-hsp20* gene silence in *A. castellanii* trophozoites by siRNA transfection. The successful transfection of *Ac-hsp20* siRNA into trophozoites was confirmed by the fluorescent staining under microscope after transfection 6 h **(A)**. The *Ac-hsp20* mRNA transcription was significantly inhibited in siRNA group compared to negative control group **(B)**. The inhibited *Ac*-HSP20 protein expression was confirmed in siRNA transfected trophozoites by Western blot with anti-*Ac*-HSP20 IgG **(C)**. Densitometric values were normalized by β-actin and expressed as mean ± *SD*, *n* = 3 **(D)**. ^*^p < 0.05.

**Figure 8:**
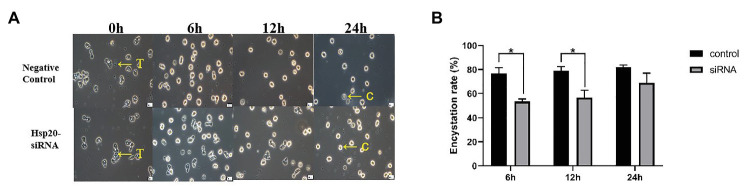
Inhibition of *A. castellanii* encystation through silencing Ac-hsp20 gene transcription in trophozoites. The trophozoites were transfected *Ac*-hsp20-siRNA and then cooled down on ice for 0, 6, 12, and 24 h. The transformation from trophozoites to cysts was observed under microscope **(A)**. The encystation rate of *A. castellanii* trophozoites at different ice incubation time points was calculated (**B**; ^*^p < 0.05). T, trophozoites; C, cysts.

The transcription of *Ac-hsp20* in *A. castellanii* trophozoites was efficiently silenced by gene-specific siRNA transfection (Figure 7). The expression of *Ac*-HSP20 was significantly inhibited at gene transcriptional level (Figure 7B) or at protein expression level (Figures 7C,D) compared to negative control (xx gene?). The encystation of *A. cantellanii* trophozoites was also significantly inhibited after the *Ac-hsp20* gene transcription was silenced by siRNA transfection (Figure 8) at the early cooling down time points (6 and 12 h), but recovered to the similar level to the control group at 24 h time point, consistent to the results of antibody inhibition. The results further suggest that *Ac*-HSP20 is essential for the encystation of *A. castellanii* trophozoites.

## Discussion

*Acanthamoeba castellanii* is a free-living protozoan, and its life cycle includes trophozoite and cyst forms. When the amoeba encounters an unfavorable living environment, the trophozoite form could quickly transform into cyst form as a self-protection mechanism. This encystation process involves massive turnover of cellular components and remodeling of organelle structure and function to produce a cryptobiotic cell with resistance to desiccation, heat, freezing, and chemical stresses (Magistrado-Coxen et al., 2019; Baig et al., 2020). However, the detail mechanism involved in the encystation of *A. castellanii* trophozoite is unknown. Identification of molecules involved in the cyst transformation is crucial for understanding the encysting process and self-protection mechanism. Blocking the process by targeting the molecules involved in the encystation could be a good approach to interrupt its life cycle because trophozoites are susceptible to chemotherapy and cysts are resistant and contribute to the recurrence and transmission of infection. In this study, a putative *Ac*-HSP20 protein was identified to be an immunodominant protein recognized by the serum from rabbit infected with *A. castellanii*, indicating that this protein was exposed to host immune system during infection. Immunolocalization assay with specific antibody revealed that the native *Ac*-HSP20 was mostly expressed on the surface of trophozoites. Further systematic study with qPCR identified that the expression of *Ac-hsp20* was gradually increased when the trophozoites were cooled down on ice and transformed to cysts (Figure 6). The expression of native *Ac*-HSP20 protein was 7.5 times higher in cysts than in trophozoites, as determined using Western blot with anti-*Ac*-HSP20 antibody (Figure 5). The predominant expression of *Ac*-HSP20 in cysts indicated its important role in the encystation of *A. castellanii*. Indeed, when *Ac*-HSP20 was neutralized by the specific anti-*Ac*-HSP0 IgG, the transformation of trophozoites to cysts was reduced at the early stage of cooling down on ice (within 12 h) compared with those without antibody incubation (*p* < 0.05, Figure 6). However, at the late stage of encystation when the protozoa were placed on ice for 24 h, most trophozoites were transformed into cysts regardless of antibody. Meanwhile, the transcriptional level of *Ac-hsp20* was significantly induced under the pressure of antibody and reached the highest level at 24 h incubation on ice (twice higher than those without antibody, Figure 6C). This finding may explain why the antibody neutralization of *Ac*-HSP20 could not offset the complete encystation of *A. castellanii* at the late stage. The antibody pressure could induce the expression of indigenous *Ac*-HSP20. When the expression of *Ac*-HSP20 overtook the antibody pressure, the encystation continued without interruption. These results may provide new clues for the parasite to escape the immune stress from its host. The inhibition of encystation of trophozoites by anti-*Ac*-HSP20 antibody was also confirmed by the silence of *Ac-hsp20* gene in trophozoites by siRNA transfection (Figure 8). All results clearly suggested that *Ac*-HSP20 is not only a housekeeping cytoplasmic protein but also a membrane protein involved in the encystation of *A. castellanii*.

*Ac*-HSP20 belongs to the HSP superfamily, and it is highly conserved during evolution (Ingolia and Craig, 1982; Verbon et al., 1992; Groenen et al., 1994). The present study demonstrated that in addition to being a chaperon protein to protect protozoa from heat shock or other environmental stress, the membrane form of *Ac*-HSP20 played a crucial biological function in the differentiation of *A. castellanii* upon harsh environmental changes as a protective mechanism. The membrane-associated sHSP family has been identified in different species of organism, including protozoa (Caspers et al., 1995; Spinner et al., 2012). It is characterized by the presence of a conserved homologous α-crystallin domain (Spinner et al., 2012). Given their surface exposure, these membrane-associated HSPs are usually immunodominant during infection. Similar to *Ac*-HSP20, the HSP20 protein of *Leishmania* is strongly recognized by sera of dogs with visceral leishmaniasis (Montagna et al., 2012). Rats infected with parasitic nematode *Strongyloides ratti* produce strong immune reactions to two sHSPs (Hombach et al., 2014). Together with the *Ac*-HSP20 reported here, all data demonstrated that sHSP family proteins were induced under stress conditions as stable chaperons and membrane functional proteins.

Considering its presence on the membrane and its critical role in the encystation of *A. castellanii*, *Ac*-HSP20 could be a good target for vaccine or drug development. Indeed, anti-*Ac*-HSP20 antibody induced macrophage-involved ADCC observed in this study, indicating that *Ac*-HSP20-induced antibody could be involved in the clearance of *A. castellanii* by macrophage phagocytosis during infection. *In-vitro* incubation of *A. castellanii* trophozoites with rabbit anti-*Ac*-HSP20 IgG significantly reduced their adhesion to or infectivity on rabbit cornea at days 14 and 28 after the infection compared with those without antibody incubation. This finding indicated that neutralizing *Ac*-HSP20 on the surface of trophozoites may reduce their infectivity to cornea. The protective immunity induced by the immunization of recombinant *Ac*-HSP20 protein against *A. castellanii* infection in the cornea of rabbit is under investigation.

## Data Availability Statement

The datasets presented in this study can be found in online repositories. The names of the repository/repositories and accession number(s) can be found in the article/supplementary material.

## Ethics Statement

The animal study was reviewed and approved by The Animal Care and Use Committee of the Jilin Medical College (approval number: 190001).

## Author Contributions

XF and WZ: conceptualization. DL, MZ, and HS: data curation. NW, DL, MZ, HS, WZ, and XF: formal analysis. XF: investigation and supervision. NW, DL, MZ, HS, and WZ: validation. NW and HS: writing – original draft. XF and WZ: writing – review and editing. All authors contributed to the article and approved the submitted version.

### Conflict of Interest

The authors declare that the research was conducted in the absence of any commercial or financial relationships that could be construed as a potential conflict of interest.
